# Application of BMSCs-coated PLGA/type I collagen composite mesh in intraperitoneal onlay mesh repair using a rat ventral incisional hernia model

**DOI:** 10.3389/fbioe.2025.1663573

**Published:** 2025-10-31

**Authors:** Mingliang He, Yi Pan, Jiayi Li, Yaqin Qi, Kangbei Zhu, Fangjie Zhang

**Affiliations:** ^1^ The Fourth School of Clinical Medicine, Zhejiang Chinese Medical University, Hangzhou, Zhejiang, China; ^2^ Department of Abdominal Wall and Hernia Surgery, Affiliated Hangzhou First People’s Hospital, School of Medicine, Westlake University, Hangzhou, Zhejiang, China; ^3^ Department of Thyroid and Breast Surgery, Hangzhou Women's Hospital, Hangzhou, China; ^4^ Key Laboratory of Integrated Traditional Chinese and Western Medicine for Biliary and Pancreatic Diseases of Zhejiang Province, Hangzhou, Zhejiang, China; ^5^ Hangzhou Institute of Digestive Diseases, Hangzhou, Zhejiang, China; ^6^ Key Laboratory of Clinical Cancer Pharmacology and Toxicology Research of Zhejiang Province, Hangzhou, Zhejiang, China

**Keywords:** IPOM, BMSCs, PLGA mesh, anti-adhesion, tissue integration

## Abstract

**Purpose:**

This study aimed to address the limitations of synthetic meshes in incisional hernia repair by developing a bioactive composite mesh combining poly(lactic-co-glycolic acid) (PLGA), type I collagen, and bone marrow mesenchymal stem cells (BMSCs).

**Methods:**

The PLGA scaffolds, fabricated via freeze-drying, were modified with collagen to enhance biocompatibility and loaded with BMSCs to promote tissue regeneration. *In vitro* and *in vivo* evaluations in a rat ventral hernia model assessed biomechanical properties, anti-adhesion efficacy, and tissue integration.

**Results:**

The PLGA-Collagen I-BMSCs mesh exhibited superior anti-adhesion performance, reduced inflammatory cell infiltration by 73.3%, and enhanced neovascularization compared to commercial meshes (Sepramesh™ and Parietex™). BMSCs modulated TGF-β1/Smad3 signaling to mitigate fibrosis, while collagen alignment improved mechanical recovery. The composite mesh degraded at a rate matching tissue regeneration, with 10% PLGA maintaining structural integrity for 20 weeks. Histological analysis revealed organized collagen deposition and minimal adhesions (Nair grade 0–1 in 100% of cases).

**Conclusion:**

These findings highlight the potential of the PLGA-Collagen I-BMSCs composite as an innovative intraperitoneal onlay mesh (IPOM) solution, offering mechanical stability, anti-adhesive properties, and regenerative bioactivity. This strategy shifts hernia repair from passive support to active tissue regeneration, providing a foundation for next-generation hernia repair materials.

## 1 Introduction

Incisional hernia (IH) represents a pathological defect that develops at the site of prior surgical incisions, attributed to compromised healing of the abdominal fascia and muscle layers. This condition leads to the protrusion of visceral organs or tissues under intra-abdominal pressure. As a common iatrogenic postoperative complication, IH exhibits an increasing global incidence, with occurrence rates reported between 4% and 10% (Sanders et al., 2023). According to the Clavien-Dindo classification system for surgical complications, IH is classified as a Grade IIIb complication (Dindo et al., 2004), requiring secondary surgical intervention. This complication indicates a significant increase in healthcare burden, alongside impaired physiological functions, and a reduced quality of life for affected patients.

The pathogenesis of IH is attributed to the complex interplay between biomechanical stress and dysregulated biological responses (Omar et al., 2023). Mechanistically, surgical trauma-induced local inflammation disrupts collagen metabolism, which is characterized by increased activity of matrix metalloproteinases (MMP-2/MMP-9) and decreased expression of tissue inhibitors of metalloproteinases (TIMP-1/TIMP-2), ultimately leading to an imbalance in extracellular matrix (ECM) synthesis and degradation (Amro et al., 2024). Concurrently, sustained mechanical tension in the abdominal wall activates the TGF-β1/Smad3 signaling pathway, promoting the aberrant differentiation of fibroblasts into myofibroblasts. This differentiation contributes to pathological scarring and a loss of fascial elasticity (Thankam et al., 2019). The resulting vicious cycle of “biological vulnerability” and “biomechanical overload” leads to a failure rate exceeding 46% for conventional suture-based repair techniques (Friedrich et al., 2008). The failure of surgical wound closure, characterized by dehiscence or recurrent herniation, is rarely attributable to a singular cause. Instead, it arises from a synergistic dysfunction involving both mechanical integrity and metabolic capacity (Omar et al., 2023; DeAngelo and Perez, 2023). Mechanically, the persistent physiological stress exerted by abdominal pressure imposes repetitive strain on the repair site, potentially leading to suture tearing, mesh dislocation, or fascia elongation. Biologically, an imbalanced matrix metalloproteinase/tissue inhibitor of metalloproteinase (MMP/TIMP) ratio and a transforming growth factor-beta 1 (TGF-β1)-driven fibrotic response contribute to a compromised local tissue environment, characterized by structurally deficient collagen, reduced vascularization, and impaired healing capacity. This metabolic dysfunction hinders the wound’s ability to withstand normal mechanical forces, while ongoing mechanical strain exacerbates the biological imbalance, perpetuating a self-sustaining cycle that ultimately culminates in the failure of the repair.

Current guidelines advocate for mesh reinforcement as the standard treatment for IH, grounded in evidence-based medicine (Hernia and Abdominal Wall Surgery Group of Chinese Society of Surgery of Chinese Medical Association, 2025). Among the various surgical techniques, intraperitoneal onlay mesh (IPOM) repair is consistent with anatomical and physiological reconstruction principles. This approach offers several advantages, including reduced operative time, minimized tissue dissection, and expedited postoperative recovery. Nonetheless, IPOM necessitates direct exposure of the mesh to the intraperitoneal environment, presenting dual challenges concerning material properties: the mesh must possess adequate mechanical strength to withstand abdominal wall tension (typically exceeding 32 N/cm) and feature a biocompatible surface to mitigate intra-abdominal adhesion (Li et al., 2022). Clinically utilized anti-adhesive meshes, such as Sepramesh™ and Parietex™ Composite Mesh, achieve short-term anti-adhesion effects through coatings comprising carboxymethyl cellulose-modified hyaluronic acid (HA-CMC), polyethylene glycol (PEG)-based gels, and collagen. In more severe cases, chronic foreign body reactions to synthetic materials can result in dense fibrous encapsulation, leading to mesh contraction, displacement, and even erosion into the bowel or bladder. A 2019 cohort study utilizing the Herniamed database, which included 9,907 cases, reported a postoperative complication rate of 5% and a reoperation rate of 2.1% following intraperitoneal onlay mesh (IPOM) procedures. The incidence of postoperative complications, including deep wound infection, bleeding, seroma, and wound healing disorders, is low and demonstrates a significant difference when compared to the open sublay technique, which is closely associated with an increased need for reoperation (Köckerling et al., 2019).

To address these challenges, this study introduces an innovative strategy involving a functionalized composite mesh. This approach employs biodegradable poly (lactic-co-glycolic acid) (PLGA) as the mechanical support framework, constructs a three-dimensional porous architecture through vacuum freeze-drying, and modifies the surface with type I collagen (Collagen I) to enhance cellular adhesion properties. Additionally, bone marrow mesenchymal stem cells (BMSCs) are loaded to create bioactive interfaces. The performance of the PLGA-Collagen I-BMSCs composite mesh in IPOM procedures is systematically evaluated using established rat abdominal wall incisional hernia models. The assessment concentrates on four principal dimensions: biomechanical properties, tissue integration efficiency, anti-adhesion efficacy, and degradation compatibility, in order to substantiate its potential for clinical translation. The results will furnish theoretical foundations and technical support for the development of innovative hernia repair materials that incorporate mechanical adaptability, bioactivity, and sustain anti-adhesion functionality.

## 2 Materials and methods

### 2.1 Fabrication of PLGA-Collagen I-BMSCs composite mesh

Poly (lactic-co-glycolic acid) (PLGA) with a lactic acid to glycolic acid molar ratio of 70:30 (molecular weight = 150 kDa, sourced from Sigma-Aldrich) underwent vacuum drying to eliminate residual moisture. Accurately measured aliquots of 5.0, 10.0, and 15.0 g were dissolved in 100 mL of dioxane (chromatographic grade, provided by Aladdin) to achieve homogeneous solutions with concentrations of 5%, 10%, and 15% (w/v), respectively. Complete dissolution of the polymer was facilitated by magnetic stirring at 800 rpm and 25 °C, in conjunction with ultrasonication at 40 kHz and 200 W for 15 min. The resulting solutions were cast into custom-fabricated polytetrafluoroethylene (PTFE) molds with dimensions of 4 × 4 × 0.1 cm^3^. These were rapidly quenched in liquid nitrogen at −196 °C for 30 s to induce instantaneous nucleation, followed by deep-freezing at −80 °C for 24 h to stabilize the ice crystal structure. Subsequently, primary drying at −50 °C and 50 Pa for 48 h, and secondary drying at 25 °C and 10 Pa for 24 h, were performed using a vacuum freeze-dryer (SP Scientific, VirTis AD2.0 EL) to produce porous PLGA scaffolds.

Type I collagen derived from bovine sources (Macklin, 3 mg/mL) was dissolved in pre-cooled 0.6% acetic acid (v/v) and subsequently infused into molds containing PLGA scaffolds. A negative pressure of −0.08 MPa was applied for a duration of 2 h to ensure uniform infiltration of collagen into the pore channels. A gradient drying process was employed, consisting of hot-air drying at 40 °C with 30% humidity for 4 h, followed by vacuum drying at 25 °C and 5 Pa for 24 h, resulting in the formation of stable PLGA-Collagen I composite structures.

Structural stability was further enhanced through glutaraldehyde vapor crosslinking. Samples were placed in sealed containers with a 0.25% glutaraldehyde solution (pH 7.4) in the upper crystallizing dishes. After 6 h of crosslinking at 37 °C, the reactions were terminated using a 0.1 M glycine solution. Residual glutaraldehyde levels were quantified using the Nash reagent method (ISO 10993), confirming concentrations of less than 0.2 μg/cm^2^. Ethylene oxide sterilization was conducted at 55 °C with 60% humidity in a 6-h cycle, and sterility was validated through sterile culture tests involving a 14-day incubation in TSB medium (Monaco et al., 2017; Wie et al., 2009; Krug et al., 2023).

Based on preliminary research and related studies, a seeding density of 1 × 10^6^ cells/cm^2^ is deemed appropriate (Hendrawan et al., 2024a; Zhang et al., 2017; Zhang, 2018). Passage 3 rat BMSCs (Procell), with a purity exceeding 98% (CD90+/CD44+/CD34-as determined by flow cytometry), were seeded onto the meshes at a density of 1 × 10^6^ cells/cm^2^. Following a 12-h incubation period at 37 °C with 5% CO_2_, PLGA-Collagen I-BMSCs composites were successfully obtained.

### 2.2 Surface characteristics of PLGA meshes

To investigate the microarchitecture of poly(lactic-co-glycolic acid) (PLGA) meshes with varying concentrations (5%, 10%, and 15%), the prepared specimens were sectioned into 5 mm × 5 mm squares and affixed onto scanning electron microscope (SEM) stubs using double-sided conductive tape, ensuring a flush contact between the sample surfaces and the stub substrates. Subsequently, the mounted specimens were subjected to gold/platinum (Au/Pt) alloy sputter-coating within a vacuum deposition system, with the coating thickness meticulously controlled to 10–20 nm to enhance electrical conductivity and mitigate charging artifacts. Following metallization, the specimen stubs were loaded into the SEM chamber and secured with mechanical clamps. The chamber was then sealed and evacuated to achieve an optimal operating vacuum (<5 × 10^−3^ Pa). The SEM was initiated with operational parameters set at an accelerating voltage of 5–20 kV. Systematic observation commenced at a low magnification (500×) for the identification of regions of interest, followed by incremental increases in magnification up to 5,000× or higher for detailed ultrastructural characterization. Digital micrographs were obtained at various magnifications, with comprehensive annotations detailing polymer concentration, magnification factors, and accelerating voltage embedded within the metadata to support subsequent morphometric analysis.

### 2.3 Degradation profile of PLGA meshes

To assess the degradation kinetics of poly(lactic-co-glycolic acid) (PLGA) meshes with varying concentrations (5%, 10%, 15%) and their collagen-crosslinked variants, pre-weighed samples were immersed in phosphate-buffered saline (PBS, pH 7.4) under controlled conditions at 37 °C. Specifically, PLGA meshes, both with and without type I collagen crosslinking, were cut into geometrically uniform squares measuring 10 × 10 × 0.1 mm^3^. Initial dry weights (W_0_) were obtained using a microbalance (Mettler Toledo XP6, with a resolution of ±0.01 mg). Each specimen was individually immersed in PBS (10 mL per sample) with sufficient spatial separation to prevent interfacial interactions, followed by incubation at 37 °C in a thermostatic shaker set at 80 rpm. On a weekly basis, the meshes were removed, rinsed three times with deionized water (18.2 MΩ cm) to remove ionic residues, and then dried to a constant weight in a vacuum oven at 60 °C (≤100 Pa, for 24 h). Post-desiccation weights (W_t_) were measured using the same microbalance. The degradation profile was quantified using:

$${Remained\;Weight}_{t}\left(\%\right)=\frac{W_{t}}{W_{0}}\times 100$$

Where W_0_ = initial dry weight, W_t_ = weight at time t.

### 2.4 Biocompatibility of PLGA meshes

Cell viability was assessed in real-time utilizing the Calcein-AM/PI Double Stain Kit (Solarbio CA1630). Composites of PLGA-Collagen I-BMSCs, 24 h post-seeding, were immersed in phosphate-buffered saline (PBS) containing 2 μM Calcein-AM and 1.5 μM propidium iodide (PI), followed by a 30-min incubation at 37 °C in the absence of light. Imaging was conducted using an inverted fluorescence microscope (Zeiss Axio Observer A1) with excitation wavelengths of 490 nm for Calcein-AM (green fluorescence) and 545 nm for PI (red fluorescence).

The CCK-8 assay (Fudebio-tech FD3788) was performed in accordance with ISO 10993-5 standards. Samples were collected at 1, 2, 3, 4 and 5 days post-seeding and were immersed in a low-serum medium (2% fetal bovine serum) containing 10% CCK-8 reagent, followed by a 2-h incubation at 37 °C. Subsequently, 100 μL of the supernatant was transferred to a 96-well plate, and the absorbance (optical density value) at 450 nm was measured using a microplate reader (SpectraMax iD5). A blank scaffold group (cell-free) served as background control, while the control group consisted of cells directly seeded onto a culture dish.

### 2.5 Mesh implantation in a rat incisional hernia repair model

Male Sprague Dawley rats (SPF grade, aged 8 weeks, with a body weight of 350 ± 20 g) were procured from the Laboratory Animal Center of Hangzhou Medical College. The animals were maintained under controlled environmental conditions, specifically at a temperature range of 22 °C–26 °C, a humidity level of 45%–65%, and a 12-h light/dark cycle, with unrestricted access to food and water. All experimental procedures adhered to the Guide for the Care and Use of Laboratory Animals.

Anesthesia was administered via intraperitoneal injection of sodium pentobarbital at a dosage of 40 mg/kg. Following the shaving and disinfection of the surgical site, a 2 cm midline longitudinal incision was made through the skin and subcutaneous tissue. Blunt dissection was conducted between the left external and internal oblique muscles to establish a 3 × 3 cm^2^ operative field. The external oblique muscle was incised longitudinally at a distance of 0.5 cm from the midline, ensuring the preservation of the rectus sheath integrity. Subsequently, a full-thickness resection of the abdominal wall tissues, including the external oblique, transversalis fascia, and peritoneum, was performed to create a 1 × 1 cm^2^ defect. Closure of the layers was accomplished using 5-0 PGA sutures (Ethicon VCP392H). Postoperative analgesia was administered using ibuprofen suspension at a dosage of 20 mg/kg/day, and infection prophylaxis was provided with cefazolin at 50 mg/kg/day subcutaneously for a duration of three consecutive days. The formation of the hernia sac was confirmed through palpation in the second week. Ultrasound imaging (LOGIQ E10, 12L linear probe) was employed to demonstrate a defect diameter of ≥0.8 cm, indicating successful modeling with a success rate of 93.2%.

Two weeks after the modeling surgery, the successfully modeled rats were then randomized into four groups (n = 6 per group): Control, Sepramesh™, Parietex™, PLGA-Collagen I, and PLGA-Collagen I-BMSCs. Under general anesthesia, the original incision was reopened, and adhesions were bluntly dissected to expose the hernia ring. Meshes were trimmed to dimensions of 2 × 2 cm^2^ and secured using non-absorbable polypropylene sutures (Ethicon PROLENE 8668H) with a four-corner suspension fixation technique (3 mm from the mesh edge, penetrating the full thickness of the abdominal wall). A tension-free coverage with a mesh overlap of ≥0.5 cm was ensured prior to the layered closure of the abdomen. The protocol for the control group mirrored that of the previously described experimental group, with the only distinction being the absence of mesh application. Multimodal evaluations conducted at postoperative week 12 included adhesion grading according to the Nair scoring system (Nair et al., 1974) (Table 1), assessment of inflammatory infiltration via H&E staining, and collagen evaluation through Masson’s trichrome staining.

**TABLE 1:** The status of intra-abdominal adhesion was assessed according to the scoring system of Nair et al.

Grade	Description of adhesive bands
0	Complete absence of adhesion
1	Single band of adhesion, between viscera or from viscera to abdominal wall
2	Twobands, either from viscera to abdominal wall
3	More than two bands, between viscera or viscera to abdominal wall
4	Viscera directly adherent to abdominal wall, irrespective of number and extent of adhesive bands

## 3 Results

### 3.1 Surface characteristics of PLGA

**FIGURE 1:**
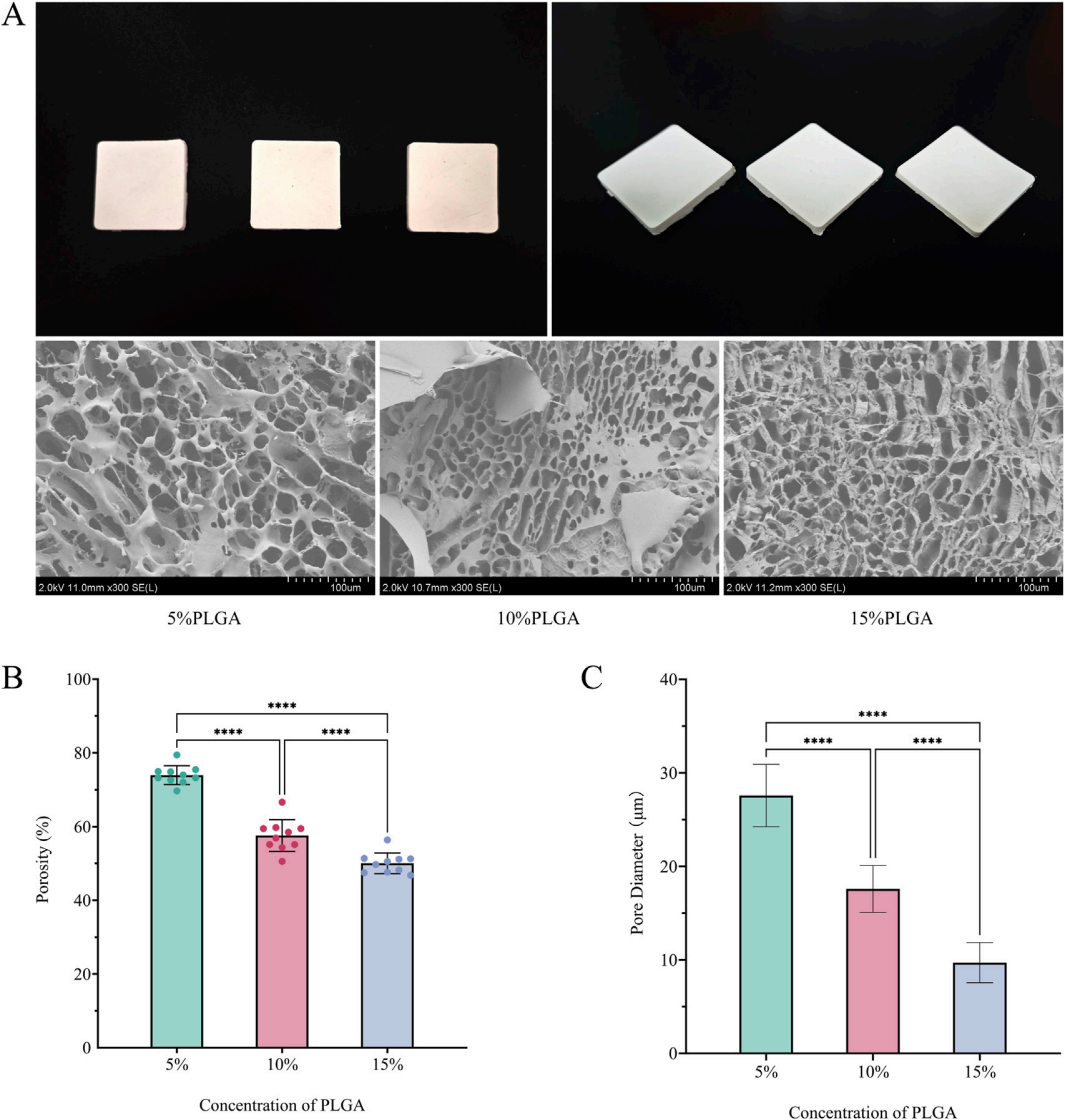
**(A)** Top view, side view, and SEM images of meshes fabricated using 5%, 10%, and 15% PLGA solutions via vacuum freeze-drying; **(B)** Porosity of meshes prepared with the three PLGA concentrations; **(C)** Pore size distribution of meshes corresponding to the three PLGA concentrations.

The application of freeze-drying technology effectively facilitated the construction of PLGA scaffolds characterized by three-dimensional interconnected porous networks, as depicted in Figure 1A. Scanning Electron Microscopy (SEM) analysis demonstrated that all scaffold groups possessed highly interconnected honeycomb-like pore structures. A significant reduction in porosity was observed with increasing concentrations of PLGA (Figure 1B): the 5% group exhibited a porosity of 74.0% ± 2.6%, the 10% group 57.6% ± 4.3%, and the 15% group 50.0% ± 2.8%, with statistical significance (p < 0.0001) confirmed by one-way ANOVA. Similarly, the pore size distribution followed a comparable trend (Figure 1C): 28 ± 3.3 μm for the 5% group, 18 ± 2.5 μm for the 10% group, and 9.7 ± 2.1 μm for the 15% group (p < 0.0001, one-way ANOVA). This porous architecture not only facilitated cell adhesion and proliferation (Walczak et al., 2017) but also provided enhanced spatial accommodation and binding sites conducive to type I collagen crosslinking. These characteristics contributed to improved collagen stability and distribution on the mesh surface, thereby promoting tissue repair and regeneration.

### 3.2 Degradation characteristics of PLGA

**FIGURE 2:**
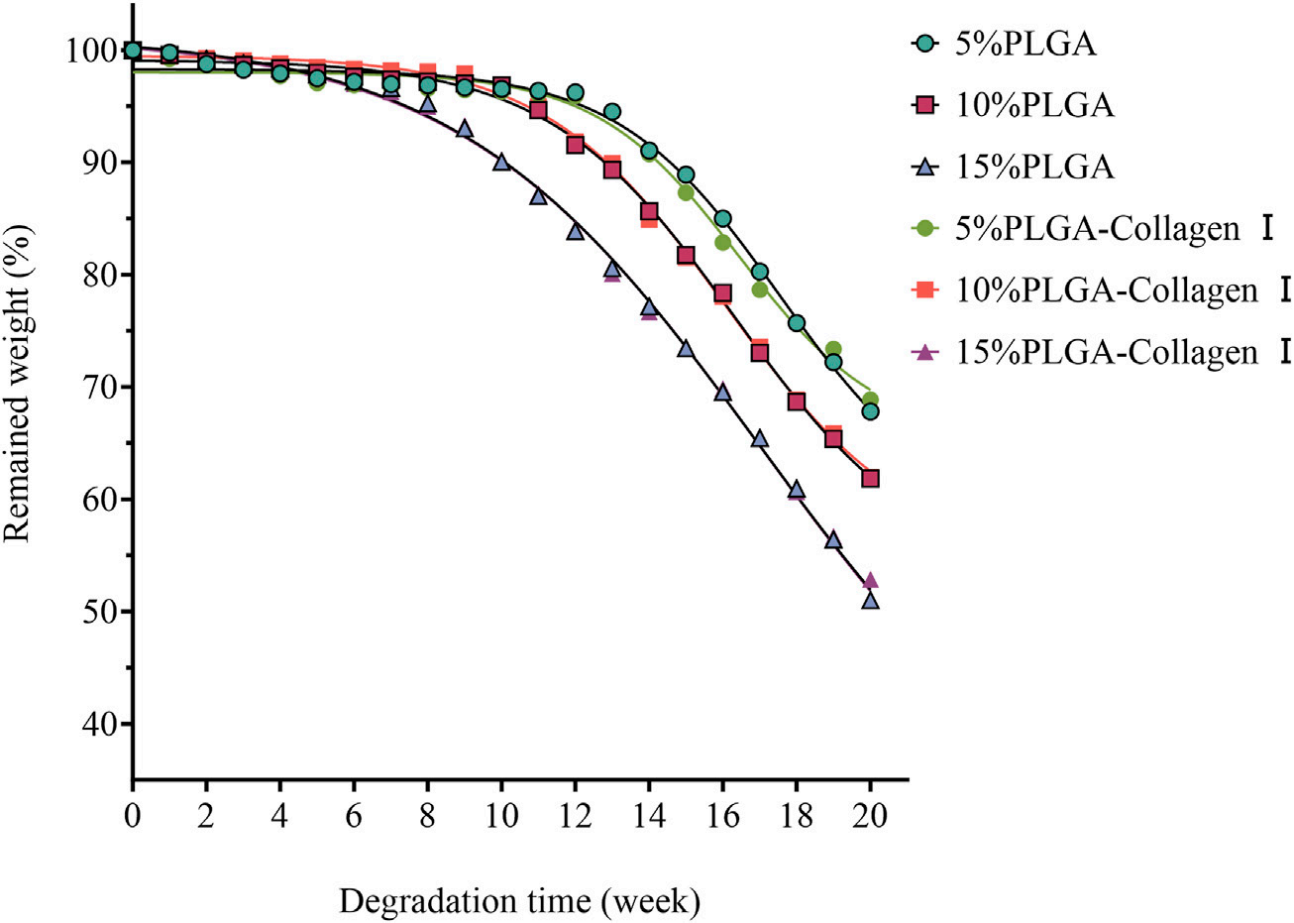
Degradation profiles of PLGA meshes (5%, 10%, 15%) and their collagen type I-crosslinked counterparts in PBS solution at 37 °C.

The PLGA-Collagen I composite mesh demonstrated distinct biphasic degradation kinetics characterized by concentration-dependent gradients, as illustrated in Figure 2. Under PBS-simulated physiological conditions, all concentration groups exhibited minimal mass loss (<5%) during the initial phase (0–4 weeks). However, degradation rates increased over time, particularly in groups with higher PLGA concentrations, which showed more significant mass reduction. Specifically, the 5% PLGA mesh experienced a slow degradation process, retaining over 95% of its mass until rapid degradation began at week 12. In contrast, the 10% and 15% PLGA meshes entered accelerated degradation phases earlier, with substantial mass loss commencing at weeks 10 and 8, respectively. This pattern indicates that higher PLGA concentrations are associated with enhanced solubility and accelerated degradation rates. Overall, PLGA degradation exhibited a concentration-dependent behavior: lower concentrations (5%) offered prolonged stability, whereas higher concentrations (10% and 15%) degraded more quickly. These findings suggest that PLGA concentration plays a critical role in modulating both the degradation rate and long-term *in vivo* stability of the composite mesh. These observations provide essential insights for the customization of mesh designs, indicating that the adjustment of PLGA concentrations can enhance tissue repair outcomes in accordance with clinical needs. Statistical analysis demonstrated no significant correlation between Collagen I crosslinking and PLGA degradation rates (p > 0.05), thereby confirming that the degradation pathways of these two components are independent. The study underscores two regulatory dimensions: the precise modulation of PLGA degradation through concentration adjustments and the crosslinking of Collagen I to modulate bioactivity, both of which facilitate the optimization of functional composite mesh performance.

### 3.3 Biocompatibility of PLGA mesh

**FIGURE 3:**
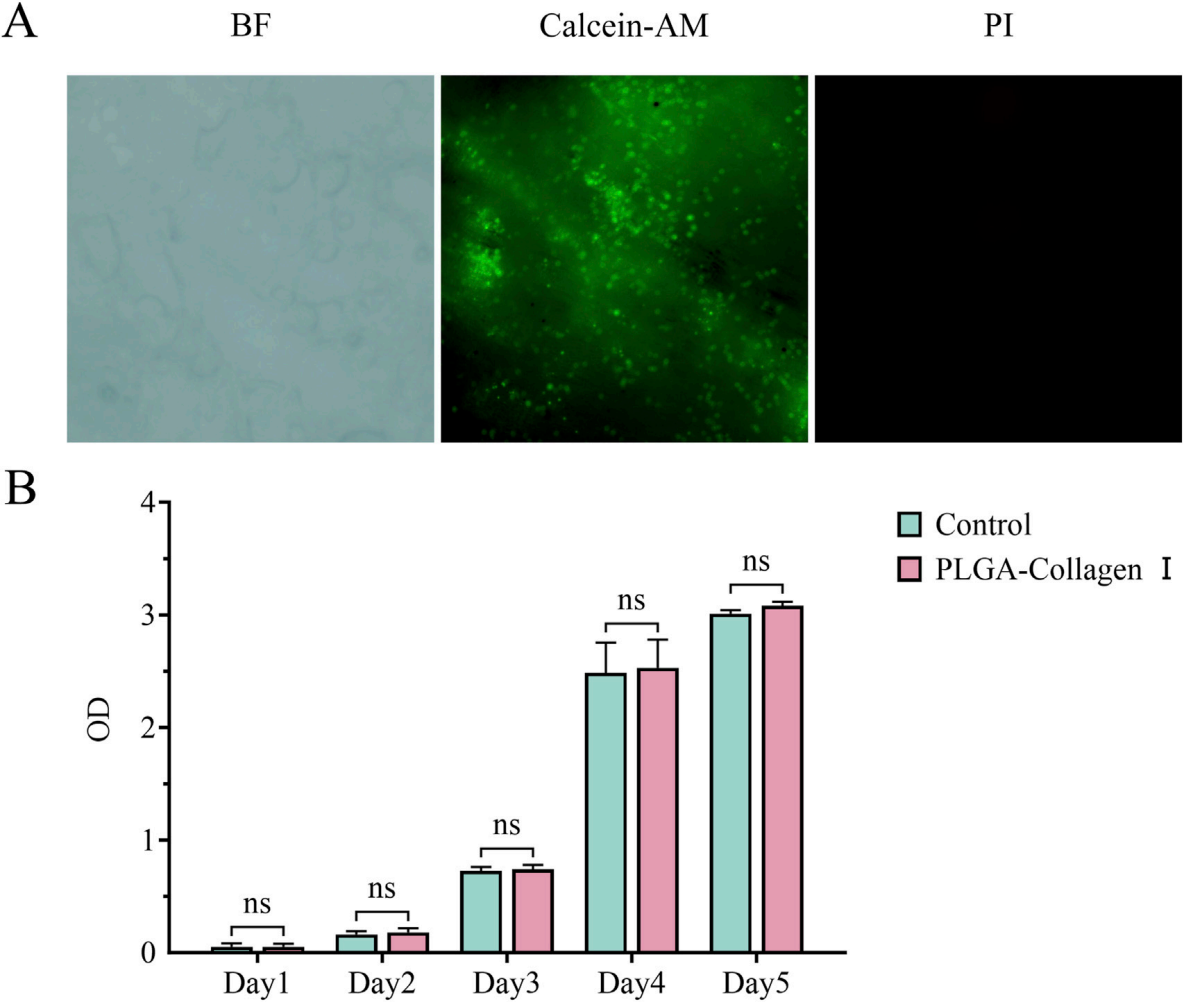
**(A)** Calcein-AM/PI fluorescence microscopy images of BMSCs seeded on PLGA-Collagen I meshes (incomplete dye removal due to porous structure); **(B)** CCK-8 assay quantifying BMSC proliferation on PLGA-Collagen I meshes (n = 5, ns (P > 0.05)).

Calcein-AM/PI dual fluorescence staining indicated a uniform distribution of dense green fluorescence signals (Calcein-AM^+^ viable cells) on the surface of the PLGA-Collagen I composite mesh at 24 h post-seeding, with a cell viability coverage of 92.4% ± 3.8% (Figure 3A). High-resolution imaging revealed that BMSCs adopted spindle or stellate morphologies along the porous scaffold, extending pseudopodia up to 48.7 ± 7.2 μm in length and establishing intercellular contacts with neighboring cells. CCK-8 assays demonstrated a sigmoidal proliferation curve for the composite mesh group (Figure 3B), with OD450 values of 0.05 ± 0.03 on day 1, entering the logarithmic growth phase (0.74 ± 0.04) by day 3, and reaching the plateau phase (3.08 ± 0.04) by day 5, showing no significant difference compared to the control group (P = 0.0766). Collectively, these findings confirm the excellent biocompatibility of PLGA, in accordance with ISO 10993-5 Grade 0 cytotoxicity criteria.

### 3.4 Mesh implantation in a rat incisional hernia repair model

**FIGURE 4:**
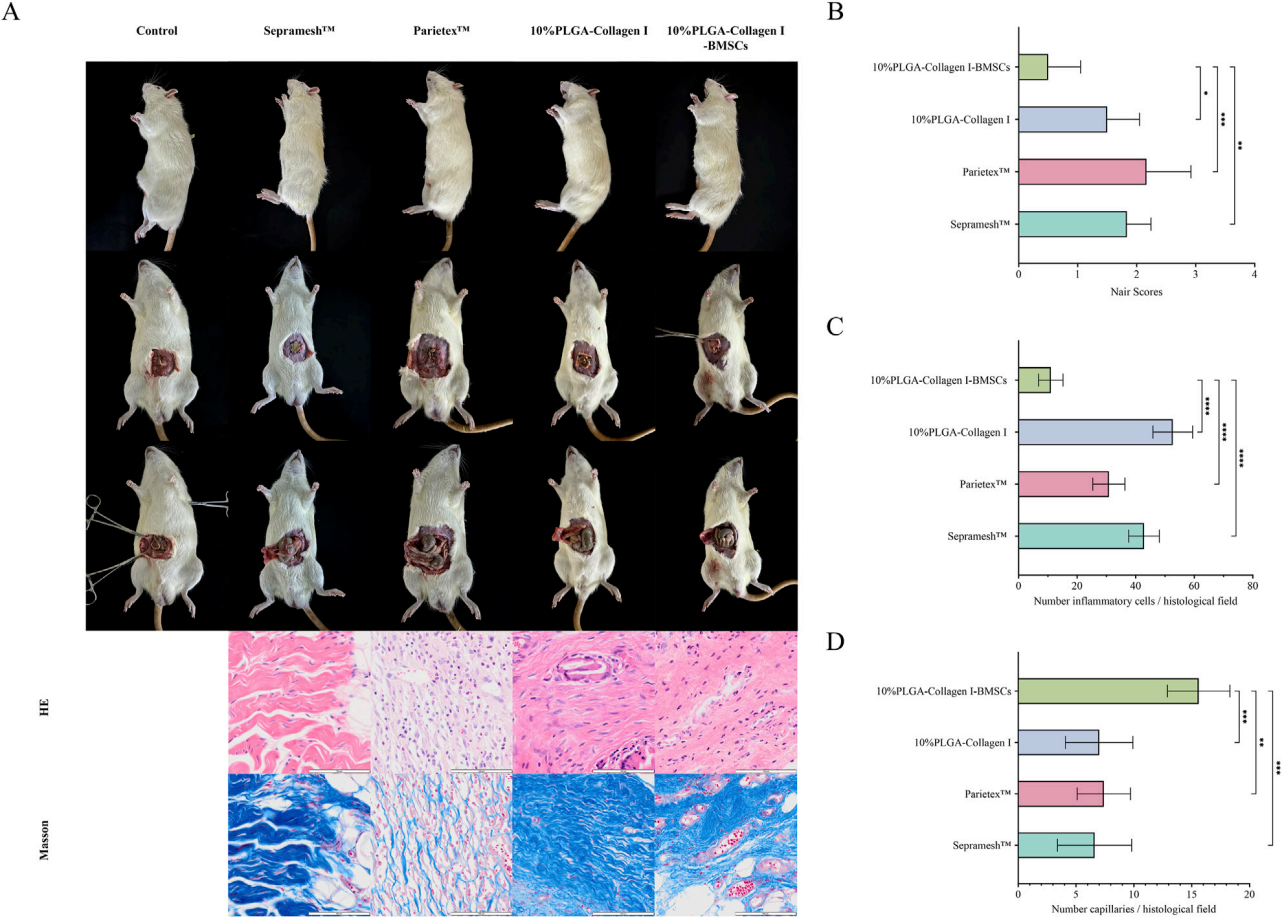
Comparative analysis at 12 weeks post-operation among groups: Control (no mesh), Sepramesh™, Parietex™, 10%PLGA-Collagen I, and 10%PLGA-Collagen I-BMSCs (n = 6). **(A)** Macroscopic abdominal wall morphology and H&E/Masson’s trichrome-stained histological sections; **(B)** Nair adhesion scores; **(C)** Inflammatory cell density; **(D)** Neovascularization density.

At 12 weeks post-operation, gross examination revealed that the PLGA-Collagen I-BMSCs mesh exhibited excellent integration with the abdominal wall tissues. Semi-transparent neo-fascial tissue enveloped the mesh margins (Figures 4A,B), and adhesion grading, assessed using the Nair scale, was significantly better than that of the control groups: Grade 0 adhesions were observed in 50.0% (3/6) of cases, Grade 1 in 50.0% (3/6), with no instances of Grade 2 or 3 adhesions. In contrast, the Parietex™ group demonstrated Grade 3 adhesions in 33.3% (2/6) of cases, while the Sepramesh™ group exhibited localized edge curling and dense fibrotic adhesions to intestinal tissues, with Grade ≥2 adhesions in 83.3% of cases. Ultrasound imaging confirmed the absence of hernia recurrence across all groups.

Histological analysis indicated that the PLGA-Collagen I-BMSCs group experienced a 73.30% reduction in inflammatory cell infiltration density (11 ± 4.2 cells/HPF) compared to the Sepramesh™ group (42.8 ± 5.2 cells/HPF, p < 0.001), with significantly lower values than the other groups (Parietex™ group: 30.8 ± 5.5 cells/HPF, 64.29% reduction, p < 0.001) (Figure 4C). The PLGA-Collagen I-BMSCs group demonstrated a significant reduction in inflammation by 79.13% compared to the BMSC-free PLGA-Collagen I group, with inflammatory cell counts of 52.7 ± 6.8 cells/HPF (p < 0.001). This group exhibited focal inflammatory foci measuring less than 0.1 mm^2^ per lesion, as opposed to the diffuse infiltration exceeding 0.5 mm^2^ observed in the control group. Masson’s trichrome staining indicated the presence of organized, dense, bundle-like collagen fibers with a highly aligned orientation in the PLGA-Collagen I-BMSCs group. Conversely, commercial meshes were characterized by disorganized collagen networks, featuring loose reticular structures and localized fiber fragmentation. The density of neovascularization at the interface between the mesh and host tissue was significantly elevated in the PLGA-Collagen I-BMSCs group, with 15.6 ± 2.7 vessels/HPF, representing a 2.2-fold increase over the PLGA-Collagen I group (7.0 ± 2.9 vessels/HPF, p < 0.001) and exceeding the values observed in commercial meshes (Sepramesh™: 6.6 ± 3.2; Parietex™: 7.4 ± 2.3 vessels/HPF, p < 0.001) (Figure 4D).

The data indicate that the loading of BMSCs not only alleviates foreign body reactions through immunomodulatory effects but also enhances functional extracellular matrix remodeling and neovascularization via paracrine mechanisms, ultimately achieving a synergistic restoration of anatomical structure and mechanical properties (Zou et al., 2023).

## 4 Discussion

This study systematically validated the comprehensive performance advantages of PLGA-Collagen I-BMSCs composite meshes in IPOM repair for abdominal incisional hernias, demonstrating their innovative value in terms of biomechanical compatibility, tissue integration efficiency, anti-adhesion mechanisms and full degradable. In comparison to traditional synthetic meshes, this composite mesh effectively addressed the challenge of balancing anti-adhesion efficacy with tissue regeneration capability through biomimetic multi-level structural design and active bio-interface engineering.

The hydrolysis process of poly(lactic-co-glycolic acid) (PLGA) can be delineated into two distinct stages. During the initial stage, the molecular weight of PLGA consistently decreases as degradation progresses, yet there is negligible weight loss. As the process transitions into the second stage, the molecular weight declines to a lower threshold and remains relatively stable despite further degradation. This behavior is characteristic of the hydrolysis of aliphatic polyesters, wherein weight loss is observed only after the molecular weight diminishes to a critical point at which the polymer becomes soluble in water. Additionally, the hydrolysis rate of PLGA porous scaffolds exhibits an inverse relationship with porosity. Prior research has demonstrated that non-porous PLGA (50/50) degrades more rapidly than foamed materials with porosities of 33% and 75%, corroborating the findings of this study (Athanasiou et al., 1998; Annaji et al., 2024; Elenskaya et al., 2024; Ghosh et al., 2023). This phenomenon is primarily ascribed to the autocatalytic effect induced by the accumulation of acidic degradation products within the polymer matrix. Scaffolds with lower porosity or larger pore sizes possess thicker pore walls and reduced specific surface areas, which inhibit the diffusion of acidic degradation products and exacerbate the local acidic environment (Wu and Ding, 2005). To achieve an optimal balance between degradation rate and mechanical strength, we selected the 10% PLGA group for subsequent investigation.

Importantly, the concentration gradient-dependent degradation behavior of PLGA allowed for precise regulation of the dynamic equilibrium between mechanical support and tissue regeneration within the mesh. In the 10% PLGA group, 61.95% of the initial mass was retained at 20 weeks, with the degradation rate closely aligning with the regeneration pace of the abdominal wall fascia. This alignment prevented both premature mechanical failure and chronic stimulation due to late-stage material residue. Furthermore, the formation of a peritoneum-like mesothelium on the surface of the mesh significantly reduced the incidence of severe intra-abdominal adhesions (Nair grade 3) from 33.3% in the Sepramesh™ group to 0%. This reduction was achieved through the dual mechanisms of physical barriers and bioactive factors (Wang et al., 2022), surpassing the limited efficacy duration of existing oxidized regenerated cellulose coatings.

Drawing upon the existing body of literature, it is reasonable to hypothesize that the PLGA-Collagen I-BMSCs composite mesh exerts a multifaceted synergistic effect in facilitating the repair process: The three-dimensional interconnected porous mesh not only provided physical pathways for cell migration (Ellermann et al., 2023; Kotlarz et al., 2023; Prakoso et al., 2023) and neovascularization (Huang et al., 2022) but also significantly enhanced the directional differentiation and functional expression of BMSCs (Liu et al., 2022) due to its high compatibility with the natural extracellular matrix (Alhosseini et al., 2012). More critically, the incorporation of BMSCs enhanced the reparative microenvironment via dual mechanisms. Firstly, BMSCs secreted anti-inflammatory factors, including interleukin-10 (IL-10) and transforming growth factor-beta 3 (TGF-β3), which significantly inhibited the polarization of macrophages towards the M1 phenotype (Cortés-Morales et al., 2023; Hu et al., 2021; Chen et al., 2022; Liu et al., 2024). This shift altered the foreign body response from a chronic inflammatory state to a pro-repair phenotype. Secondly, BMSCs directly mitigated the excessive activation of the TGF-β1/Smad3 signaling pathway in fibroblasts (Kim et al., 2018) by delivering regulatory molecules such as miR-29b and miR-210 via exosomes (Zhang et al., 2017; Zheng et al., 2022; Guo et al., 2024), thereby reducing pathological scar formation (Feng et al., 2022; Xu et al., 2022). This synergistic effect of immune regulation and paracrine signaling was evidenced by Masson’s trichrome staining, which revealed an orderly collagen arrangement aligned with the mechanical conduction direction of the abdominal wall muscles (Zhang et al., 2024). This alignment potentially decreases the risk of mechanical tearing at the mesh edge through stress-shielding effects. BMSCs facilitated capillary formation via paracrine mechanisms, including the secretion of platelet-derived growth factor-C, vascular endothelial growth factor, and angiopoietin-like protein 4 (Zhou et al., 2023; Aquino et al., 2021), in addition to direct contact mechanisms (Chen et al., 2021; Méndez-Barbero et al., 2021). This dual approach enhanced the microenvironment by supplying essential oxygen, nutrients, specific hormones, and growth factors necessary for tissue repair (Li et al., 2020). The biofunctionalization of mesh materials represents a prominent area of contemporary research. The study conducted by Siufui Hendrawan and colleagues illustrates that approaches such as the incorporation of human umbilical cord mesenchymal stem cells (hUC-MSCs) into prosthetic meshes or their exposure to bioactive treatments can significantly enhance tissue healing and regeneration following hernia repair (Hendrawan et al., 2024a; Hendrawan et al., 2024b), thereby demonstrating considerable potential for practical application.

While this study used commercially sourced rats for consistency and reproducibility, effectively translating the PLGA-Collagen I-MSCs composite meshes for human use requires careful selection of the best cell source. MSCs are primarily sourced from adipose tissue (via liposuction or lipectomy), umbilical cord tissue (especially Wharton’s jelly and blood vessels), and bone marrow (usually from the iliac bone and crest). The two main strategies are autologous (patient-derived) and allogeneic (donor-derived) MSCs, each with distinct benefits, logistical challenges, and regulatory considerations (At de et al., 2024). Autologous MSCs offer perfect immunocompatibility, eliminating rejection risks and allowing long-term engraftment without immunosuppression. However, their clinical use faces challenges: a, A 3–6 weeks delay for isolation and preparation, unsuitable for urgent hernia repairs; b. Reduced potency in elderly, diabetic, or obese patients, who are more prone to hernias; c. High costs and infrastructure needs due to GMP compliance, limiting accessibility (Cunnane et al., 2018). Allogeneic MSCs from young, healthy donors provide a practical solution for large-scale clinical use. Clinical evidence shows that these cells, with low MHC-II expression and lacking co-stimulatory molecules, have strong immunomodulatory properties and low immunogenicity, rarely facing rejection. They evade immune detection through mechanisms like IDO secretion, Treg induction, and HLA-G expression. Allogeneic MSCs offer consistent potency, immediate availability, and lower costs due to scalable production (Fan et al., 2020). For MSCs used to modulate inflammation and promote regeneration, allogeneic MSCs are a more viable short-term clinical option due to their transient presence.

Nonetheless, this study presents several limitations: firstly, the intra-abdominal pressure in rat models, ranging from 0 to 5 mmHg, is considerably lower than that in humans, potentially leading to an overestimation of the mesh’s long-term mechanical stability. As the PLGA degrades, it undergoes changes in mechanical strength, complicating the assessment of its dynamic variations during the repair process. Secondly, the paracrine lineage of BMSCs and their interaction mechanisms with host immune cells necessitate further investigation through single-cell sequencing and other omics technologies. Furthermore, this applied research did not directly quantify or verify the differentiation capacity or functional expression of BMSCs when seeded onto 3D PLGA scaffolds through *in vitro* experiments. Thirdly, the local pH fluctuations resulting from mesh degradation products have not been quantitatively evaluated for their impact on peritoneal integrity. Future research should focus on developing large animal models, such as those involving pigs, with abdominal wall defects and conducting multicenter randomized controlled trials to assess the clinical translation potential of this material.

In conclusion, the PLGA-Collagen I-BMSCs composite mesh presents an innovative approach to the anatomical and functional dual repair of abdominal incisional hernias through an integrated design strategy characterized by “structural biomimicry-functional activation-degradation adaptation.” The primary contribution of this study lies in its shift from a passive repair paradigm to an active regenerative medicine approach, thereby establishing both theoretical and practical foundations for the development of next-generation intelligent hernia repair materials.

## Data Availability

The raw data supporting the conclusions of this article will be made available by the authors, without undue reservation.
